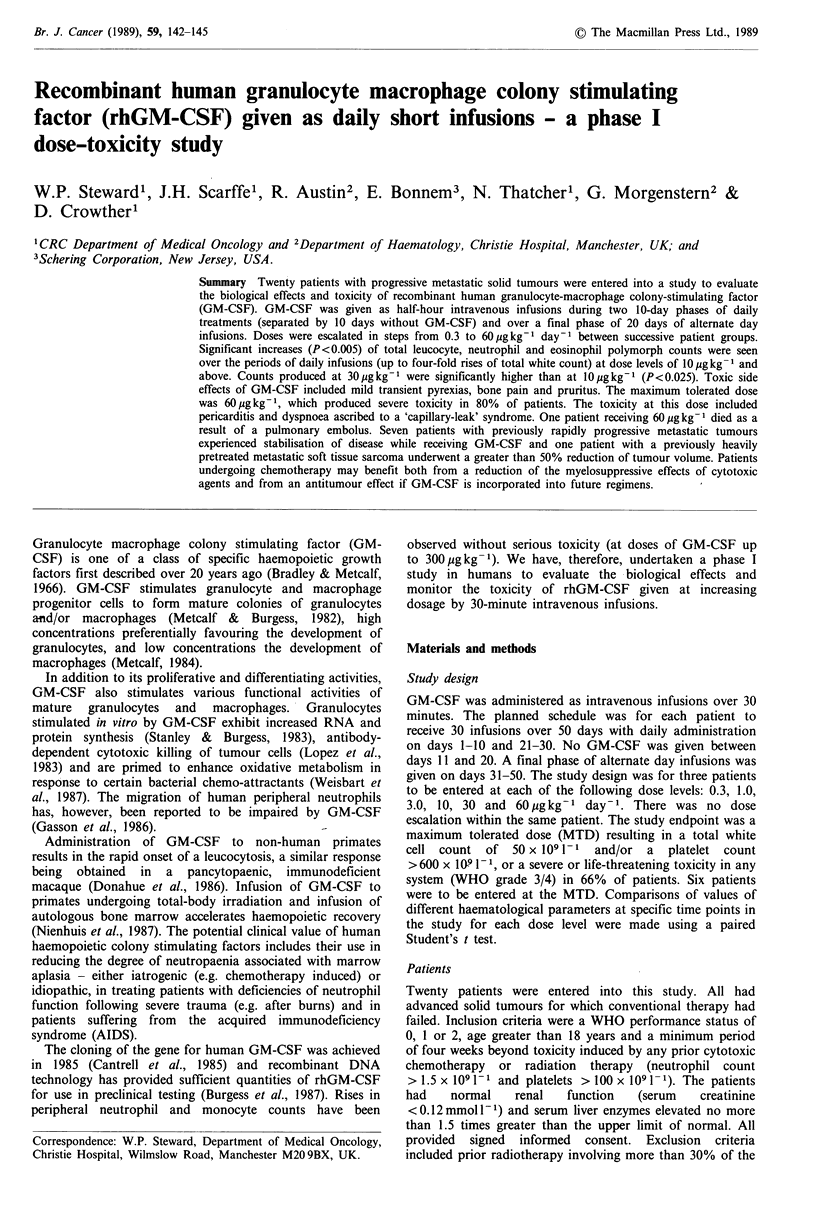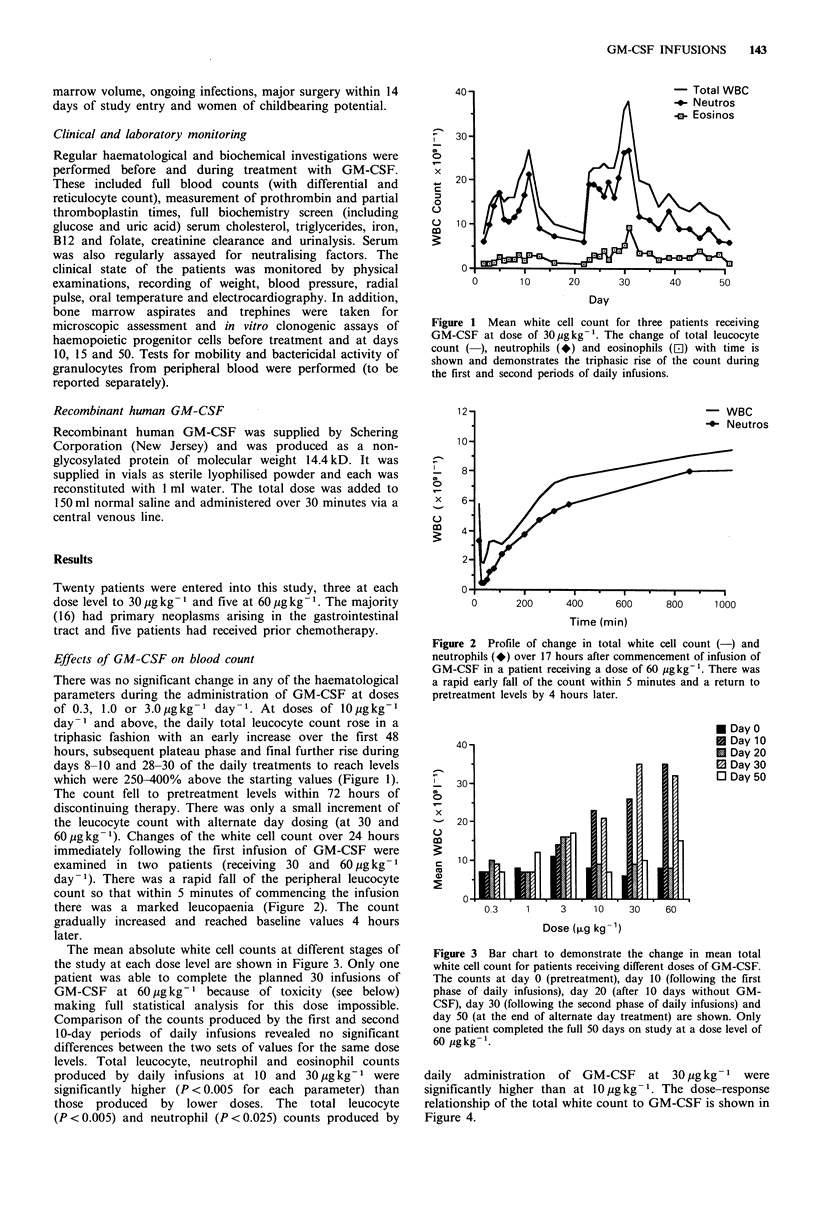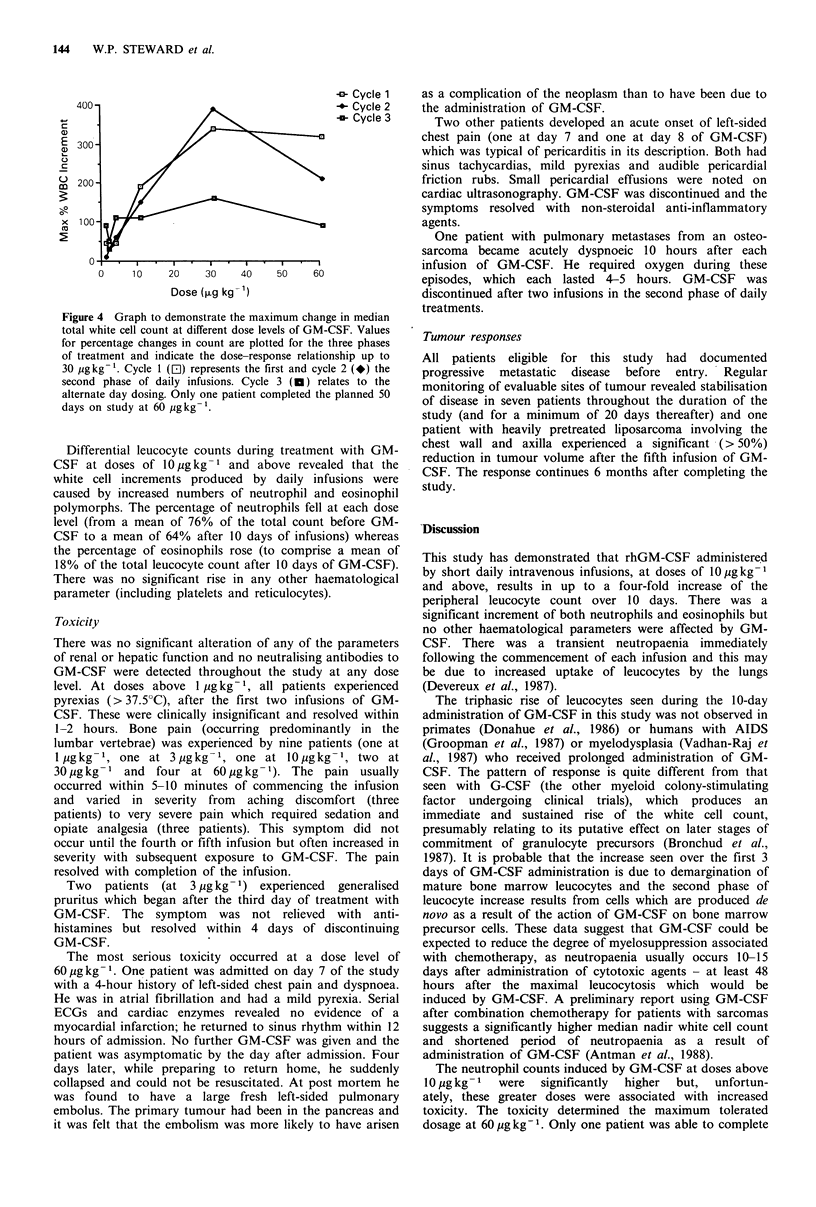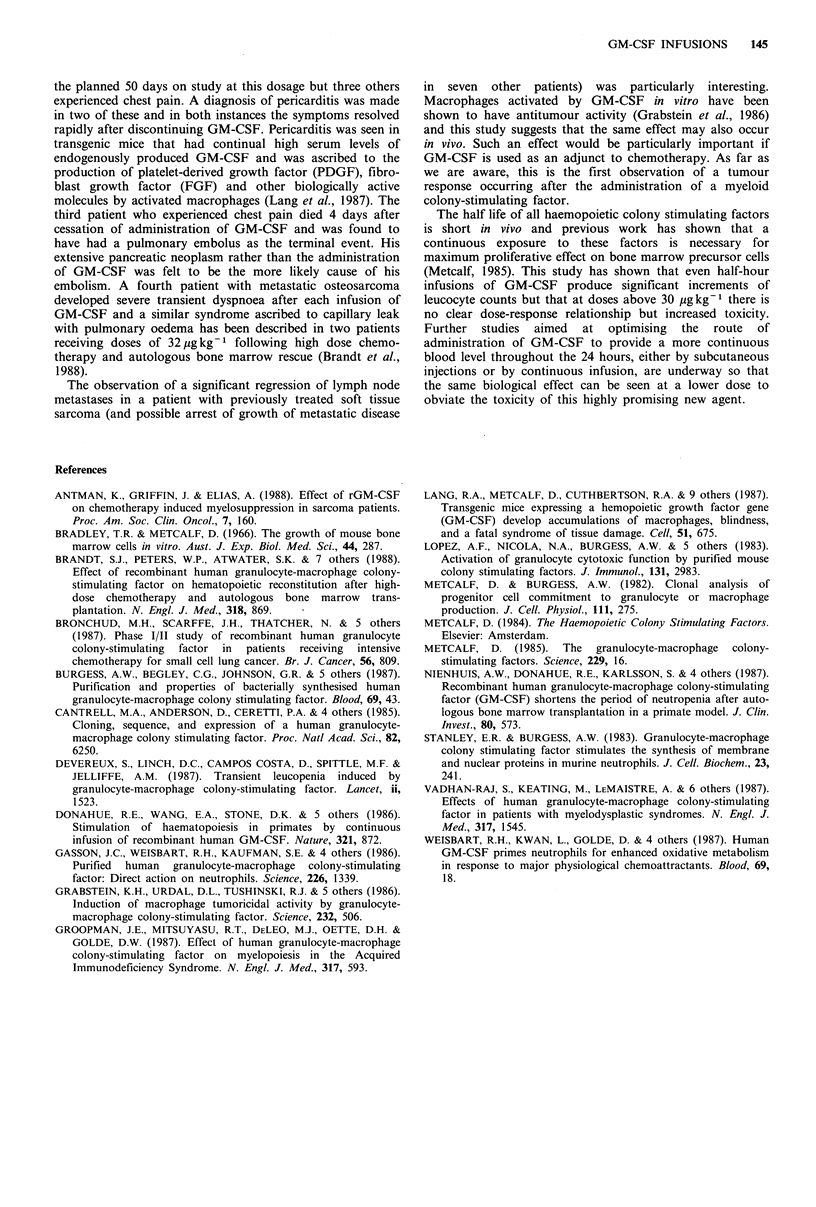# Recombinant human granulocyte macrophage colony stimulating factor (rhGM-CSF) given as daily short infusions--a phase I dose-toxicity study.

**DOI:** 10.1038/bjc.1989.28

**Published:** 1989-01

**Authors:** W. P. Steward, J. H. Scarffe, R. Austin, E. Bonnem, N. Thatcher, G. Morgenstern, D. Crowther

**Affiliations:** CRC Department of Medical Oncology, Christie Hospital, Manchester, UK.

## Abstract

Twenty patients with progressive metastatic solid tumours were entered into a study to evaluate the biological effects and toxicity of recombinant human granulocyte-macrophage colony-stimulating factor (GM-CSF). GM-CSF was given as half-hour intravenous infusions during two 10-day phases of daily treatments (separated by 10 days without GM-CSF) and over a final phase of 20 days of alternate day infusions. Doses were escalated in steps from 0.3 to 60 micrograms kg-1 day-1 between successive patient groups. Significant increases (P less than 0.005) of total leucocyte, neutrophil and eosinophil polymorph counts were seen over the periods of daily infusions (up to four-fold rises of total white count) at dose levels of 10 micrograms kg-1 and above. Counts produced at 30 micrograms kg-1 were significantly higher than at 10 micrograms kg-1 (P less than 0.025). Toxic side effects of GM-CSF included mild transient pyrexias, bone pain and pruritus. The maximum tolerated dose was 60 micrograms kg-1, which produced severe toxicity in 80% of patients. The toxicity at this dose included pericarditis and dyspnoea ascribed to a 'capillary-leak' syndrome. One patient receiving 60 micrograms kg-1 died as a result of a pulmonary embolus. Seven patients with previously rapidly progressive metastatic tumours experienced stabilisation of disease while receiving GM-CSF and one patient with a previously heavily pretreated metastatic soft tissue sarcoma underwent a greater than 50% reduction of tumour volume. Patients undergoing chemotherapy may benefit both from a reduction of the myelosuppressive effects of cytotoxic agents and from an antitumour effect if GM-CSF is incorporated into future regimens.


Br. J. Cancer (1989), 59, 142–145 © The Macmillan Press Ltd., 1989

### Recombinant human granulocyte macrophage colony stimulating factor (rhGM-CSF) given as daily short infusions – a phase I dose–toxicity study

W.P. Steward[1], J.H. Scarffe[1], R. Austin[2], E. Bonnem[3], N. Thatcher[1], G. Morgenstern[2] & D. Crowther[1]

[1]*CRC Department of Medical Oncology and* [2]*Department of Haematology, Christie Hospital, Manchester, UK; and* [3]*Schering Corporation, New Jersey, USA.*

**Summary** Twenty patients with progressive metastatic solid tumours were entered into a study to evaluate the biological effects and toxicity of recombinant human granulocyte-macrophage colony-stimulating factor (GM-CSF). GM-CSF was given as half-hour intravenous infusions during two 10-day phases of daily treatments (separated by 10 days without GM-CSF) and over a final phase of 20 days of alternate day infusions. Doses were escalated in steps from 0.3 to $60\,\mu g\,kg^{-1}\,day^{-1}$ between successive patient groups. Significant increases ($P<0.005$) of total leucocyte, neutrophil and eosinophil polymorph counts were seen over the periods of daily infusions (up to four-fold rises of total white count) at dose levels of $10\,\mu g\,kg^{-1}$ and above. Counts produced at $30\,\mu g\,kg^{-1}$ were significantly higher than at $10\,\mu g\,kg^{-1}$ ($P<0.025$). Toxic side effects of GM-CSF included mild transient pyrexias, bone pain and pruritus. The maximum tolerated dose was $60\,\mu g\,kg^{-1}$, which produced severe toxicity in 80% of patients. The toxicity at this dose included pericarditis and dyspnoea ascribed to a 'capillary-leak' syndrome. One patient receiving $60\,\mu g\,kg^{-1}$ died as a result of a pulmonary embolus. Seven patients with previously rapidly progressive metastatic tumours experienced stabilisation of disease while receiving GM-CSF and one patient with a previously heavily pretreated metastatic soft tissue sarcoma underwent a greater than 50% reduction of tumour volume. Patients undergoing chemotherapy may benefit both from a reduction of the myelosuppressive effects of cytotoxic agents and from an antitumour effect if GM-CSF is incorporated into future regimens.

Granulocyte macrophage colony stimulating factor (GM-CSF) is one of a class of specific haemopoietic growth factors first described over 20 years ago (Bradley & Metcalf, 1966). GM-CSF stimulates granulocyte and macrophage progenitor cells to form mature colonies of granulocytes and/or macrophages (Metcalf & Burgess, 1982), high concentrations preferentially favouring the development of granulocytes, and low concentrations the development of macrophages (Metcalf, 1984).

In addition to its proliferative and differentiating activities, GM-CSF also stimulates various functional activities of mature granulocytes and macrophages. Granulocytes stimulated *in vitro* by GM-CSF exhibit increased RNA and protein synthesis (Stanley & Burgess, 1983), antibody-dependent cytotoxic killing of tumour cells (Lopez *et al.*, 1983) and are primed to enhance oxidative metabolism in response to certain bacterial chemo-attractants (Weisbart *et al.*, 1987). The migration of human peripheral neutrophils has, however, been reported to be impaired by GM-CSF (Gasson *et al.*, 1986).

Administration of GM-CSF to non-human primates results in the rapid onset of a leucocytosis, a similar response being obtained in a pancytopaenic, immunodeficient macaque (Donahue *et al.*, 1986). Infusion of GM-CSF to primates undergoing total-body irradiation and infusion of autologous bone marrow accelerates haemopoietic recovery (Nienhuis *et al.*, 1987). The potential clinical value of human haemopoietic colony stimulating factors includes their use in reducing the degree of neutropaenia associated with marrow aplasia – either iatrogenic (e.g. chemotherapy induced) or idiopathic, in treating patients with deficiencies of neutrophil function following severe trauma (e.g. after burns) and in patients suffering from the acquired immunodeficiency syndrome (AIDS).

The cloning of the gene for human GM-CSF was achieved in 1985 (Cantrell *et al.*, 1985) and recombinant DNA technology has provided sufficient quantities of rhGM-CSF for use in preclinical testing (Burgess *et al.*, 1987). Rises in peripheral neutrophil and monocyte counts have been observed without serious toxicity (at doses of GM-CSF up to $300\,\mu g\,kg^{-1}$). We have, therefore, undertaken a phase I study in humans to evaluate the biological effects and monitor the toxicity of rhGM-CSF given at increasing dosage by 30-minute intravenous infusions.

Correspondence: W.P. Steward, Department of Medical Oncology, Christie Hospital, Wilmslow Road, Manchester M20 9BX, UK.

### Materials and methods

### *Study design*

GM-CSF was administered as intravenous infusions over 30 minutes. The planned schedule was for each patient to receive 30 infusions over 50 days with daily administration on days 1–10 and 21–30. No GM-CSF was given between days 11 and 20. A final phase of alternate day infusions was given on days 31–50. The study design was for three patients to be entered at each of the following dose levels: 0.3, 1.0, 3.0, 10, 30 and $60\,\mu g\,kg^{-1}\,day^{-1}$. There was no dose escalation within the same patient. The study endpoint was a maximum tolerated dose (MTD) resulting in a total white cell count of $50\times10^9\,l^{-1}$ and/or a platelet count $>600\times10^9\,l^{-1}$, or a severe or life-threatening toxicity in any system (WHO grade 3/4) in 66% of patients. Six patients were to be entered at the MTD. Comparisons of values of different haematological parameters at specific time points in the study for each dose level were made using a paired Student's *t* test.

### *Patients*

Twenty patients were entered into this study. All had advanced solid tumours for which conventional therapy had failed. Inclusion criteria were a WHO performance status of 0, 1 or 2, age greater than 18 years and a minimum period of four weeks beyond toxicity induced by any prior cytotoxic chemotherapy or radiation therapy (neutrophil count $>1.5\times10^9\,l^{-1}$ and platelets $>100\times10^9\,l^{-1}$). The patients had normal renal function (serum creatinine $<0.12\,mmol\,l^{-1}$) and serum liver enzymes elevated no more than 1.5 times greater than the upper limit of normal. All provided signed informed consent. Exclusion criteria included prior radiotherapy involving more than 30% of the marrow volume, ongoing infections, major surgery within 14 days of study entry and women of childbearing potential.

*Clinical and laboratory monitoring*

Regular haematological and biochemical investigations were performed before and during treatment with GM-CSF. These included full blood counts (with differential and reticulocyte count), measurement of prothrombin and partial thromboplastin times, full biochemistry screen (including glucose and uric acid) serum cholesterol, triglycerides, iron, B12 and folate, creatinine clearance and urinalysis. Serum was also regularly assayed for neutralising factors. The clinical state of the patients was monitored by physical examinations, recording of weight, blood pressure, radial pulse, oral temperature and electrocardiography. In addition, bone marrow aspirates and trephines were taken for microscopic assessment and *in vitro* clonogenic assays of haemopoietic progenitor cells before treatment and at days 10, 15 and 50. Tests for mobility and bactericidal activity of granulocytes from peripheral blood were performed (to be reported separately).

*Recombinant human GM-CSF*

Recombinant human GM-CSF was supplied by Schering Corporation (New Jersey) and was produced as a non-glycosylated protein of molecular weight 14.4 kD. It was supplied in vials as sterile lyophilised powder and each was reconstituted with 1 ml water. The total dose was added to 150 ml normal saline and administered over 30 minutes via a central venous line.

### Results

Twenty patients were entered into this study, three at each dose level to 30 $\mu$g kg$^{-1}$ and five at 60 $\mu$g kg$^{-1}$. The majority (16) had primary neoplasms arising in the gastrointestinal tract and five patients had received prior chemotherapy.

*Effects of GM-CSF on blood count*

There was no significant change in any of the haematological parameters during the administration of GM-CSF at doses of 0.3, 1.0 or 3.0 $\mu$g kg$^{-1}$ day$^{-1}$. At doses of 10 $\mu$g kg$^{-1}$ day$^{-1}$ and above, the daily total leucocyte count rose in a triphasic fashion with an early increase over the first 48 hours, subsequent plateau phase and final further rise during days 8–10 and 28–30 of the daily treatments to reach levels which were 250–400% above the starting values (Figure 1). The count fell to pretreatment levels within 72 hours of discontinuing therapy. There was only a small increment of the leucocyte count with alternate day dosing (at 30 and 60 $\mu$g kg$^{-1}$). Changes of the white cell count over 24 hours immediately following the first infusion of GM-CSF were examined in two patients (receiving 30 and 60 $\mu$g kg$^{-1}$ day$^{-1}$). There was a rapid fall of the peripheral leucocyte count so that within 5 minutes of commencing the infusion there was a marked leucopaenia (Figure 2). The count gradually increased and reached baseline values 4 hours later.

The mean absolute white cell counts at different stages of the study at each dose level are shown in Figure 3. Only one patient was able to complete the planned 30 infusions of GM-CSF at 60 $\mu$g kg$^{-1}$ because of toxicity (see below) making full statistical analysis for this dose impossible. Comparison of the counts produced by the first and second 10-day periods of daily infusions revealed no significant differences between the two sets of values for the same dose levels. Total leucocyte, neutrophil and eosinophil counts produced by daily infusions at 10 and 30 $\mu$g kg$^{-1}$ were significantly higher ($P < 0.005$ for each parameter) than those produced by lower doses. The total leucocyte ($P < 0.005$) and neutrophil ($P < 0.025$) counts produced by daily administration of GM-CSF at 30 $\mu$g kg$^{-1}$ were significantly higher than at 10 $\mu$g kg$^{-1}$. The dose–response relationship of the total white count to GM-CSF is shown in Figure 4.

**Figure 1** Mean white cell count for three patients receiving GM-CSF at dose of 30 $\mu$g kg$^{-1}$. The change of total leucocyte count (—), neutrophils (◆) and eosinophils (▣) with time is shown and demonstrates the triphasic rise of the count during the first and second periods of daily infusions.

**Figure 2** Profile of change in total white cell count (—) and neutrophils (◆) over 17 hours after commencement of infusion of GM-CSF in a patient receiving a dose of 60 $\mu$g kg$^{-1}$. There was a rapid early fall of the count within 5 minutes and a return to pretreatment levels by 4 hours later.

**Figure 3** Bar chart to demonstrate the change in mean total white cell count for patients receiving different doses of GM-CSF. The counts at day 0 (pretreatment), day 10 (following the first phase of daily infusions), day 20 (after 10 days without GM-CSF), day 30 (following the second phase of daily infusions) and day 50 (at the end of alternate day treatment) are shown. Only one patient completed the full 50 days on study at a dose level of 60 $\mu$g kg$^{-1}$.

**Figure 4** Graph to demonstrate the maximum change in median total white cell count at different dose levels of GM-CSF. Values for percentage changes in count are plotted for the three phases of treatment and indicate the dose–response relationship up to 30 $\mu$g kg$^{-1}$. Cycle 1 (□) represents the first and cycle 2 (◆) the second phase of daily infusions. Cycle 3 (■) relates to the alternate day dosing. Only one patient completed the planned 50 days on study at 60 $\mu$g kg$^{-1}$.

Differential leucocyte counts during treatment with GM-CSF at doses of 10 $\mu$g kg$^{-1}$ and above revealed that the white cell increments produced by daily infusions were caused by increased numbers of neutrophil and eosinophil polymorphs. The percentage of neutrophils fell at each dose level (from a mean of 76% of the total count before GM-CSF to a mean of 64% after 10 days of infusions) whereas the percentage of eosinophils rose (to comprise a mean of 18% of the total leucocyte count after 10 days of GM-CSF). There was no significant rise in any other haematological parameter (including platelets and reticulocytes).

*Toxicity*

There was no significant alteration of any of the parameters of renal or hepatic function and no neutralising antibodies to GM-CSF were detected throughout the study at any dose level. At doses above 1 $\mu$g kg$^{-1}$, all patients experienced pyrexias (> 37.5°C), after the first two infusions of GM-CSF. These were clinically insignificant and resolved within 1–2 hours. Bone pain (occurring predominantly in the lumbar vertebrae) was experienced by nine patients (one at 1 $\mu$g kg$^{-1}$, one at 3 $\mu$g kg$^{-1}$, one at 10 $\mu$g kg$^{-1}$, two at 30 $\mu$g kg$^{-1}$ and four at 60 $\mu$g kg$^{-1}$). The pain usually occurred within 5–10 minutes of commencing the infusion and varied in severity from aching discomfort (three patients) to very severe pain which required sedation and opiate analgesia (three patients). This symptom did not occur until the fourth or fifth infusion but often increased in severity with subsequent exposure to GM-CSF. The pain resolved with completion of the infusion.

Two patients (at 3 $\mu$g kg$^{-1}$) experienced generalised pruritus which began after the third day of treatment with GM-CSF. The symptom was not relieved with anti-histamines but resolved within 4 days of discontinuing GM-CSF.

The most serious toxicity occurred at a dose level of 60 $\mu$g kg$^{-1}$. One patient was admitted on day 7 of the study with a 4-hour history of left-sided chest pain and dyspnoea. He was in atrial fibrillation and had a mild pyrexia. Serial ECGs and cardiac enzymes revealed no evidence of a myocardial infarction; he returned to sinus rhythm within 12 hours of admission. No further GM-CSF was given and the patient was asymptomatic by the day after admission. Four days later, while preparing to return home, he suddenly collapsed and could not be resuscitated. At post mortem he was found to have a large fresh left-sided pulmonary embolus. The primary tumour had been in the pancreas and it was felt that the embolism was more likely to have arisen as a complication of the neoplasm than to have been due to the administration of GM-CSF.

Two other patients developed an acute onset of left-sided chest pain (one at day 7 and one at day 8 of GM-CSF) which was typical of pericarditis in its description. Both had sinus tachycardias, mild pyrexias and audible pericardial friction rubs. Small pericardial effusions were noted on cardiac ultrasonography. GM-CSF was discontinued and the symptoms resolved with non-steroidal anti-inflammatory agents.

One patient with pulmonary metastases from an osteo-sarcoma became acutely dyspnoeic 10 hours after each infusion of GM-CSF. He required oxygen during these episodes, which each lasted 4–5 hours. GM-CSF was discontinued after two infusions in the second phase of daily treatments.

*Tumour responses*

All patients eligible for this study had documented progressive metastatic disease before entry. Regular monitoring of evaluable sites of tumour revealed stabilisation of disease in seven patients throughout the duration of the study (and for a minimum of 20 days thereafter) and one patient with heavily pretreated liposarcoma involving the chest wall and axilla experienced a significant (> 50%) reduction in tumour volume after the fifth infusion of GM-CSF. The response continues 6 months after completing the study.

### Discussion

This study has demonstrated that rhGM-CSF administered by short daily intravenous infusions, at doses of 10 $\mu$g kg$^{-1}$ and above, results in up to a four-fold increase of the peripheral leucocyte count over 10 days. There was a significant increment of both neutrophils and eosinophils but no other haematological parameters were affected by GM-CSF. There was a transient neutropaenia immediately following the commencement of each infusion and this may be due to increased uptake of leucocytes by the lungs (Devereux *et al.*, 1987).

The triphasic rise of leucocytes seen during the 10-day administration of GM-CSF in this study was not observed in primates (Donahue *et al.*, 1986) or humans with AIDS (Groopman *et al.*, 1987) or myelodysplasia (Vadhan-Raj *et al.*, 1987) who received prolonged administration of GM-CSF. The pattern of response is quite different from that seen with G-CSF (the other myeloid colony-stimulating factor undergoing clinical trials), which produces an immediate and sustained rise of the white cell count, presumably relating to its putative effect on later stages of commitment of granulocyte precursors (Bronchud *et al.*, 1987). It is probable that the increase seen over the first 3 days of GM-CSF administration is due to demargination of mature bone marrow leucocytes and the second phase of leucocyte increase results from cells which are produced *de novo* as a result of the action of GM-CSF on bone marrow precursor cells. These data suggest that GM-CSF could be expected to reduce the degree of myelosuppression associated with chemotherapy, as neutropaenia usually occurs 10–15 days after administration of cytotoxic agents – at least 48 hours after the maximal leucocytosis which would be induced by GM-CSF. A preliminary report using GM-CSF after combination chemotherapy for patients with sarcomas suggests a significantly higher median nadir white cell count and shortened period of neutropaenia as a result of administration of GM-CSF (Antman *et al.*, 1988).

The neutrophil counts induced by GM-CSF at doses above 10 $\mu$g kg$^{-1}$ were significantly higher but, unfortunately, these greater doses were associated with increased toxicity. The toxicity determined the maximum tolerated dosage at 60 $\mu$g kg$^{-1}$. Only one patient was able to complete the planned 50 days on study at this dosage but three others experienced chest pain. A diagnosis of pericarditis was made in two of these and in both instances the symptoms resolved rapidly after discontinuing GM-CSF. Pericarditis was seen in transgenic mice that had continual high serum levels of endogenously produced GM-CSF and was ascribed to the production of platelet-derived growth factor (PDGF), fibroblast growth factor (FGF) and other biologically active molecules by activated macrophages (Lang *et al.*, 1987). The third patient who experienced chest pain died 4 days after cessation of administration of GM-CSF and was found to have had a pulmonary embolus as the terminal event. His extensive pancreatic neoplasm rather than the administration of GM-CSF was felt to be the more likely cause of his embolism. A fourth patient with metastatic osteosarcoma developed severe transient dyspnoea after each infusion of GM-CSF and a similar syndrome ascribed to capillary leak with pulmonary oedema has been described in two patients receiving doses of $32\,\mu g\,kg^{-1}$ following high dose chemotherapy and autologous bone marrow rescue (Brandt *et al.*, 1988).

The observation of a significant regression of lymph node metastases in a patient with previously treated soft tissue sarcoma (and possible arrest of growth of metastatic disease in seven other patients) was particularly interesting. Macrophages activated by GM-CSF *in vitro* have been shown to have antitumour activity (Grabstein *et al.*, 1986) and this study suggests that the same effect may also occur *in vivo*. Such an effect would be particularly important if GM-CSF is used as an adjunct to chemotherapy. As far as we are aware, this is the first observation of a tumour response occurring after the administration of a myeloid colony-stimulating factor.

The half life of all haemopoietic colony stimulating factors is short *in vivo* and previous work has shown that a continuous exposure to these factors is necessary for maximum proliferative effect on bone marrow precursor cells (Metcalf, 1985). This study has shown that even half-hour infusions of GM-CSF produce significant increments of leucocyte counts but that at doses above $30\,\mu g\,kg^{-1}$ there is no clear dose-response relationship but increased toxicity. Further studies aimed at optimising the route of administration of GM-CSF to provide a more continuous blood level throughout the 24 hours, either by subcutaneous injections or by continuous infusion, are underway so that the same biological effect can be seen at a lower dose to obviate the toxicity of this highly promising new agent.

### References

ANTMAN, K., GRIFFIN, J. & ELIAS, A. (1988). Effect of rGM-CSF on chemotherapy induced myelosuppression in sarcoma patients. *Proc. Am. Soc. Clin. Oncol.*, **7**, 160.

BRADLEY, T.R. & METCALF, D. (1966). The growth of mouse bone marrow cells *in vitro*. *Aust. J. Exp. Biol. Med. Sci.*, **44**, 287.

BRANDT, S.J., PETERS, W.P., ATWATER, S.K. & 7 others (1988). Effect of recombinant human granulocyte-macrophage colony-stimulating factor on hematopoietic reconstitution after high-dose chemotherapy and autologous bone marrow transplantation. *N. Engl. J. Med.*, **318**, 869.

BRONCHUD, M.H., SCARFFE, J.H., THATCHER, N. & 5 others (1987). Phase I/II study of recombinant human granulocyte colony-stimulating factor in patients receiving intensive chemotherapy for small cell lung cancer. *Br. J. Cancer*, **56**, 809.

BURGESS, A.W., BEGLEY, C.G., JOHNSON, G.R. & 5 others (1987). Purification and properties of bacterially synthesised human granulocyte-macrophage colony stimulating factor. *Blood*, **69**, 43.

CANTRELL, M.A., ANDERSON, D., CERETTI, P.A. & 4 others (1985). Cloning, sequence, and expression of a human granulocyte-macrophage colony stimulating factor. *Proc. Natl Acad. Sci.*, **82**, 6250.

DEVEREUX, S., LINCH, D.C., CAMPOS COSTA, D., SPITTLE, M.F. & JELLIFFE, A.M. (1987). Transient leucopenia induced by granulocyte-macrophage colony-stimulating factor. *Lancet*, **ii**, 1523.

DONAHUE, R.E., WANG, E.A., STONE, D.K. & 5 others (1986). Stimulation of haematopoiesis in primates by continuous infusion of recombinant human GM-CSF. *Nature*, **321**, 872.

GASSON, J.C., WEISBART, R.H., KAUFMAN, S.E. & 4 others (1986). Purified human granulocyte-macrophage colony-stimulating factor: Direct action on neutrophils. *Science*, **226**, 1339.

GRABSTEIN, K.H., URDAL, D.L., TUSHINSKI, R.J. & 5 others (1986). Induction of macrophage tumoricidal activity by granulocyte-macrophage colony-stimulating factor. *Science*, **232**, 506.

GROOPMAN, J.E., MITSUYASU, R.T., DELEO, M.J., OETTE, D.H. & GOLDE, D.W. (1987). Effect of human granulocyte-macrophage colony-stimulating factor on myelopoiesis in the Acquired Immunodeficiency Syndrome. *N. Engl. J. Med.*, **317**, 593.

LANG, R.A., METCALF, D., CUTHBERTSON, R.A. & 9 others (1987). Transgenic mice expressing a hemopoietic growth factor gene (GM-CSF) develop accumulations of macrophages, blindness, and a fatal syndrome of tissue damage. *Cell*, **51**, 675.

LOPEZ, A.F., NICOLA, N.A., BURGESS, A.W. & 5 others (1983). Activation of granulocyte cytotoxic function by purified mouse colony stimulating factors. *J. Immunol.*, **131**, 2983.

METCALF, D. & BURGESS, A.W. (1982). Clonal analysis of progenitor cell commitment to granulocyte or macrophage production. *J. Cell. Physiol.*, **111**, 275.

METCALF, D. (1984). *The Haemopoietic Colony Stimulating Factors*. Elsevier: Amsterdam.

METCALF, D. (1985). The granulocyte-macrophage colony-stimulating factors. *Science*, **229**, 16.

NIENHUIS, A.W., DONAHUE, R.E., KARLSSON, S. & 4 others (1987). Recombinant human granulocyte-macrophage colony-stimulating factor (GM-CSF) shortens the period of neutropenia after autologous bone marrow transplantation in a primate model. *J. Clin. Invest.*, **80**, 573.

STANLEY, E.R. & BURGESS, A.W. (1983). Granulocyte-macrophage colony stimulating factor stimulates the synthesis of membrane and nuclear proteins in murine neutrophils. *J. Cell. Biochem.*, **23**, 241.

VADHAN-RAJ, S., KEATING, M., LEMAISTRE, A. & 6 others (1987). Effects of human granulocyte-macrophage colony-stimulating factor in patients with myelodysplastic syndromes. *N. Engl. J. Med.*, **317**, 1545.

WEISBART, R.H., KWAN, L., GOLDE, D. & 4 others (1987). Human GM-CSF primes neutrophils for enhanced oxidative metabolism in response to major physiological chemoattractants. *Blood*, **69**, 18.